# Emergence and Spread of *Chlamydia trachomatis* Variant, Sweden

**DOI:** 10.3201/eid1409.080153

**Published:** 2008-09

**Authors:** Björn Herrmann, Anna Törner, Nicola Low, Markus Klint, Anders Nilsson, Inga Velicko, Thomas Söderblom, Anders Blaxhult

**Affiliations:** Uppsala University Hospital, Uppsala, Sweden (B. Herrmann, M. Klint, A. Nilsson); Swedish Institute for Infectious Disease Control, Solna, Sweden (A.Törner, I. Velicko, T. Söderblom, A. Blaxhult); University of Bern, Bern, Switzerland (N. Low)

**Keywords:** *Chlamydia trachomatis*, Sweden, genetic variant, diagnostics, PCR, infectious diseases, epidemiology, surveillance, dispatch

## Abstract

A variant of *Chlamydia trachomatis* that had escaped detection by commonly used systems was discovered in Sweden in 2006. In a nationwide study, we found that it is now prevalent across Sweden, irrespective of the detection system used. Genetic analysis by multilocus sequence typing identified a predominant variant, suggesting recent emergence.

In 2006 a new variant of *Chlamydia trachomatis* (nvCT) was discovered in Sweden (*1*). Because of a 377-bp deletion in the target sequence for amplification, the variant had escaped detection by 2 widely used nucleic acid amplification tests, Abbott m2000 (Abbott Laboratories, Abbott Park, IL, USA) and Cobas Amplicor/TaqMan48 (Roche Diagnostics, Basel, Switzerland) (*1*,*2*). The other test commonly used in Sweden, ProbeTec ET (Becton Dickinson [BD], Franklin Lakes, NJ, USA), detects the new variant because it uses a different DNA target sequence in the cryptic plasmid (*3*). The nvCT has now been reported from several of Sweden’s 21 counties (Figure 1). The aim of this study was to provide a national overview of the characteristics and extent of the new chlamydia variant through examination of surveillance trends, microbiologic laboratory data, and genetic analysis of new variant strains.

**Figure 1:**
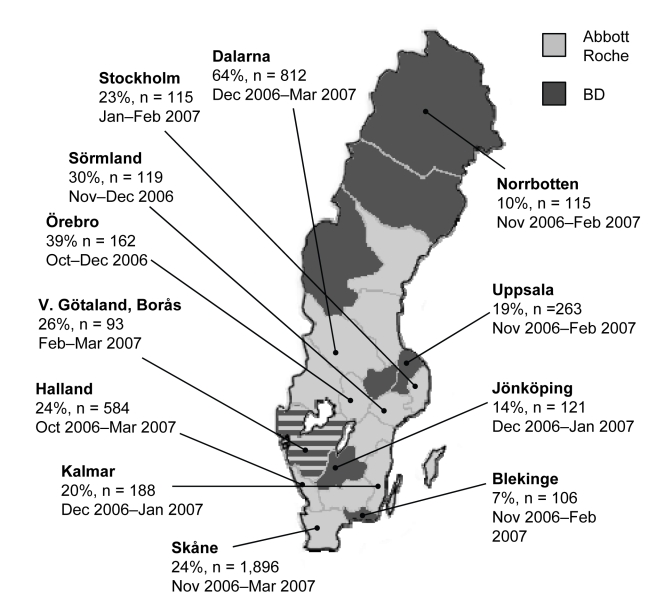
Map of Sweden showing proportions of the new variant of *Chlamydia trachomatis* in different counties. Light gray shading indicates counties that used Abbott or Roche test systems before the discovery of the new variant; dark gray shading indicates counties that used the Becton Dickinson (BD) system. The 1 county that used both Roche and BD assays is indicated with stripes. n, number of positive chlamydia cases analyzed. The period in which samples were collected is given for each county.

## The Study

We examined national surveillance data reported to the Swedish Institute for Infectious Disease Control (Smittskyddsinstitutet) about chlamydia cases detected and the number of chlamydia tests performed. Trend analysis from 2004 to 2006 included only data for the first 6 months of each year to avoid any influence of changes in detection systems; some counties introduced different detection systems in late 2006 in response to the emergence of nvCT. The statistical methods are described in the Technical Appendix. The total number of chlamydia cases detected in Sweden in the first 6 months of 2006 was lower than that in 2005, and the proportion of tests that were positive also fell (Table 1). In 2004, the proportion of positive chlamydia tests was similar whether laboratories used Abbott/Roche or BD test systems. From 2004 to 2005, there was a relative reduction of 3.4% (95% confidence interval [CI] 5.8–1.0) in chlamydia positivity in laboratories using the Abbott or Roche methods (p = 0.006) but no change in the proportion of positive samples in laboratories using the BD test system (–0.4%, 95% CI –4.2 to +3.5). During the first 6 months of 2005 and 2006, the positivity rates of samples tested by Abbott or Roche systems fell further; samples tested that used the BD system remained stable. The estimated difference in proportions of chlamydia-positive samples in counties that used Abbott or Roche tests compared with counties that used the BD method was –9.5 % (95% CI –14.1 to –4.7, p = 0.0005), after baseline differences and county differences in testing were controlled for.

**Table 1:** Numbers of chlamydia tests, positive results, and change in positivity rates, Sweden, 2004–2006

Diagnostic test*	No. counties	No. positive results/total no. tests (%) in first 6 mo		
		2004	2005	2006
Abbott/Roche	14	11,721/150,080 (7.8)	11,111/147,311 (7.5)	10,236/152,960 (6.7)
Becton Dickinson	8	4,262/54,260 (7.9)	5,220/66,728 (7.8)	3,363/43,189 (7.8)
All tests	21	15,983/204,340 (7.8)	16,331/214,039 (7.6)	13,599/196,149 (6.9)

*One county used both Roche and Becton Dickinson tests.

We conducted microbiologic analyses on consecutive samples that were collected from 12 counties in late 2006 and early 2007. Cases of nvCT were identified by testing specimens with additional methods using alternative sequence targets (Technical Appendix). The proportion of nvCT ranged from 20% to 64% in the 8 counties that used Abbott or Roche detection systems, compared with 7% to 19%, respectively, for counties that used BD tests (Figure 1). Additional data about gender, age, and clinical setting of diagnoses were available for 600 chlamydia-positive patients in the 4 counties using the BD system (Table 2). The proportion of nvCT cases varied between clinics (p = 0.020) and was higher at youth and venereal disease clinics than at antenatal and gynecology clinics. This variance might be because persons seeking treatment in these settings have higher levels of risk taking and more frequent changes in partners (*4*). There was no evidence of differences in the proportion of cases by gender (p = 0.103) or age (p = 0.558) because of nvCT.

**Table 2:** Distribution of cases of nvCT by gender, age, and clinic category in 4 selected counties that used the Becton Dickinson system*

Factor	% nvCT (n/N)	p value†
Gender		
Male	16 (45/273)	0.103
Female	12 (39/327)	
Age, y		
15–19	14 (23/162)	0.558
20–24	16 (44/279)	
25–29	11 (11/98)	
>30	10 (6/59)	
Clinic		
Venereal disease	15 (19/129)	0.020
Youth clinics	19 (39/210)	
Gynecology	9 (13/150)	
Antenatal/general practice	8 (6/77)	
Others	21 (7/34)	

*nvCT, new variant of *Chlamydia trachomatis*; n, number of nvCT cases detected; N, total number of *C. trachomatis* cases detected. †Determined by χ^2^ test.

Genetic characterization with a new high-resolution genotyping system (*5*) was performed on 48 specimens of nvCT from 2 counties that used the BD test system (n = 21); 2 counties that used Roche and Abbott systems (n = 18); and 9 specimens from Norway, Ireland, and France (Technical Appendix). The nvCT showed a new genetic variant in the chromosomal target region *hctB* compared with previous findings in wild-type strains and thus constitutes a separate clone with the designation 21 (hctB), 19 (CT058), 1 (CT144), 2 (CT172), and 1 (pbpB) in our system. All 48 specimens tested were of genotype E, and 46 were identical in the *ompA* gene to the reference strain E/Bour. The divergent specimens were from 2 persons known to be sexual partners and differed in a single nucleotide position. In the other 5 target genes, altogether comprising some 5,500 bp, all 48 specimens were identical.

## Conclusions

Our study was a national systematic overview including surveillance, demographic, microbiologic, and genetic data about the emergence and spread of a mutant strain of *C. trachomatis* in Sweden. A fall in the proportion of positive chlamydia test results in counties using Abbott or Roche test systems began in 2005 and continued in 2006, whereas positivity rates in counties using BD tests did not change. The mutant strain has now spread throughout Sweden. Notably, the new variant has scarcely been found outside Sweden (*6*), indicating that we need to improve our understanding of the sexual networks through which chlamydiae spread (*7*).

Our analysis suggests that widespread transmission of nvCT is recent, even if the mutation itself occurred some time ago, because 46 of 48 specimens from different places had identical sequences when we used a highly discriminatory multilocus sequence typing system (*5*). This hypothesis is supported by the lack of diversification in the mutant strains compared with the high degree of sequence variation in other sample collections that we have analyzed (*5*; and unpub. data). We expect new nucleotide substitutions to occur over time.

The area in Sweden in which nvCT originated is not known, but the consistently high proportion of nvCT found in the county of Dalarna suggests that the mutant might have been present in this region for longer than in other counties studied. nvCT comprised 64% of chlamydia-positive specimens over the study period in Dalarna as a whole, and up to 78% in some localities (*7*); elsewhere in Sweden (*8*–*10*) and worldwide (*11*,*12*), genotype E strains of different subvariants comprise ≈40% of chlamydia-positive speciments in heterosexual populations. These data might indicate that the high proportion of nvCT is not only a result of accumulation of chlamydia cases when diagnostics failed and treatment and contact tracing were inadequate. Further studies will be needed to determine whether nvCT also has a selective advantage that might outcompete the wild-type bacterium over time.

**Figure 2:**
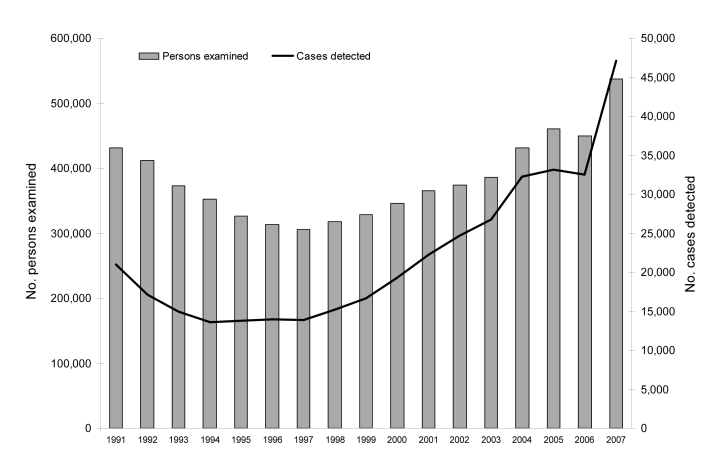
*Chlamydia trachomatis* reports, Sweden, 1991–2007. The number of persons examined and cases detected in 2007, when diagnostic tests for chlamydia had been changed, is in line with the increasing trend from 2004 and before. The figures for 2005 and 2006 reflect the failure to detect cases of the new chlamydia variant in some counties.

The emergence of this mutant strain of a sexually transmitted pathogen has implications for public health practice. A recent study estimates that some 8,000 chlamydia cases escaped detection in 2006 (*7*). This would have resulted in an ≈20% increase in reported chlamydia cases. Actual national figures for 2007 confirm such an increase, and the number of reported chlamydia cases has reached an all-time high in Sweden (Figure 2). *Chlamydia* infections, caused not only by nvCT, continue to rise (*13*,*14*), but the areas most heavily affected by the spread of nvCT have been in much the same situation as before chlamydia was first recognized as a pathogen. Failure to detect the nvCT over time have resulted in episodes of complicated infection all over the country, leading to ectopic pregnancies and infertility. Research is now needed to determine whether sequelae associated with chlamydia occur disproportionately in counties where test systems fail to diagnose the nvCT. The ability of this new variant to escape detection for so long shows that developers of future diagnostic tests need to take into account the structure and function of genomes when selecting appropriate target nucleic acid sequences in microorganisms. Currently, there are unique opportunities for research that could lead to insights into the immunobiology, transmission, and consequences of *C. trachomatis*.

## Supplementary Material

Technical Appendix Emergence and Spread of Chlamydia trachomatis Variant, Sweden
