# Supplementary material for: Emergence and Spread of Chlamydia trachomatis Variant, Sweden
Source: Emerg Infect Dis. 2008 Sep;14(9):1462–5. doi: 10.3201/eid1409.080153 (PMC2603114; doi:10.3201/eid1409.080153)
Supplement: Technical Appendix — Emergence and Spread of Chlamydia trachomatis Variant, Sweden [file 08-0153_Techapp-s1.pdf]

Publisher: CDC; Journal: Emerging Infectious Diseases
Article Type: Dispatch; Volume: 14; Issue: 9; Year: 2008; Article ID: 08-0153
DOI: 10.3201/eid/1409.080153; TOC Head: Dispatch

Emergence and Spread of *Chlamydia trachomatis* Variant, Sweden

Technical Appendix

Analysis of National Surveillance Data

We used reports of diagnosed chlamydia infections reported to the Swedish Institute for Infectious Disease Control for the years 2004, 2005, and 2006. In late 2006, the institute recommended changes in laboratory diagnostic test systems to counties using Abbott (m2000) or Roche (COBAS Amplicor/TaqMan 48 CT ver 1) tests as a result of the new variant *Chlamydia trachomatis* (nvCT, see below). Because this could have affected patterns of testing and diagnosis, we restricted our analysis to data for the first 6 months of each year. First, we compared aggregated data from all counties from the first 6 months of 2004 and 2005, and calculated the relative change (with 95% confidence intervals [CI]) in the proportions of positive samples in counties using Abbott or Roche tests, and counties using the Becton Dickinson (BD) test. We then used a random effects model to examine the association between the diagnostic method and change in positivity rate between the first 6 months of 2005 and 2006. The model allowed for adjustment for the underlying time trend from 2005 to 2006, differences in positivity rates already present in 2005, and for county specific differences in coverage in testing of the target population. Examination of residual plots showed a good fit for the model.

Microbiologic Analysis

We collected data from consecutive samples tested over a period of 2 to 4 months in 12 counties between October 2006 and March 2007. From October 2006, all counties that used Abbott or Roche test systems for routine chlamydia diagnostics tested specimens in parallel with an assay using a target sequence not in the cryptic plasmid but in the chromosomal *ompA* gene (Artus *C. trachomatis* PCR, Qiagen, Hilden, Germany, or LightMix 480HT, TIB Molbiol, Berlin, Germany) (*1*), following a recommendation from the Swedish Institute for Infectious Disease Control. Discrepant cases were regarded as the new *C. trachomatis* variant.

Confirmation of nvCT was performed on subsets of discrepant cases by means of a real-time PCR specific to nvCT (*2*). In 4 counties (Norrbotten, Uppsala, Jönköping and Blekinge) that used the BD system for routine testing, consecutive chlamydia-positive specimens were collected from late November 2006 to February 2007 Urine samples were concentrated by centrifugation for 10 min at 5000 *g* and swab samples by Microcon centrifugation (Millipore) before DNA was purified using the MagAttract DNA mini kit (QIAGEN). The purified samples were reanalyzed with the Roche COBAS TaqMan 48 CT test. All specimens displaying discrepant results were tested by the PCR assay specific to nvCT (*2*). For each county we calculated the proportion of chlamydia positive specimens caused by nvCT. For the 4 counties that provided individual level data we compared the distribution of nvCT and wild type chlamydia cases according to gender, age, and diagnostic setting using chi-square tests.

Genetic Characterization

We used a new high-resolution genotyping system (*3*) to analyze specimens taken between October 2006 and March 2007. Specimens of nvCT were from 2 counties (Norrbotten, n = 7 and Uppsala, n = 14), which used the BD test system and from the counties Örebro, n = 13, and Skåne n = 5, which used the Roche and Abbott system, respectively. In addition, we analyzed 6 samples from Norway, 2 from Ireland and 1 from France. Accession numbers of sequences describing genetic variants found in the 48 nvCT specimens examined: CT058 type 19: EF690305; CT144 type 1: EF690306; CT172 type 2: EF690307; *hctB* type 21: EF690308; *pbpB* type 1: EF690309; *ompA*: EF690310 and X52557.

References

1. Unemo M, Olcén P, Agné-Stadling I, Feldt A, Jurstrand M, Herrmann B, et al. Experiences with the new genetic variant of *Chlamydia trachomatis* in Örebro county, Sweden—proportion, characteristics and effective diagnostic solution in an emergent situation. Euro Surveill. 2007;12:699.

2. Ripa T, Nilsson PA. A *Chlamydia trachomatis* strain with a 377-bp deletion in the cryptic plasmid causing false-negative nucleic acid amplification tests. Sex Transm Dis. 2007;34:255–6.

3. Klint M, Fuxelius HH, Goldkuhl RR, Skarin H, Rutemark C, Andersson SG, et al. High-Resolution Genotyping of *Chlamydia trachomatis* Strains by Multilocus Sequence Analysis. J Clin Microbiol. 2007;45:1410–4.
